# Umbilical Artery Catheter-Associated Perianal Ischemia in a Preterm Infant

**DOI:** 10.7759/cureus.107611

**Published:** 2026-04-23

**Authors:** Jeremy B Altman, Dakota Poschel, David Zabel

**Affiliations:** 1 Department of Plastic and Reconstructive Surgery, Augusta University, Medical College of Georgia, Augusta, USA

**Keywords:** internal pudendal artery, neonatal limb ischemia, perianal necrosis, premature infant, umbilical artery catheter

## Abstract

Severe ischemic complications following umbilical arterial catheterization (UAC) in preterm neonates are well recognized but almost invariably occur during placement or while the catheter remains in situ. We report a male infant born at 25 weeks’ gestation in whom bilateral crescent-shaped zones of full-thickness perianal necrosis (each ≈3 × 1 cm) developed within hours of uncomplicated removal of a correctly positioned UAC. This report describes severe, isolated perianal necrosis developing within hours of uneventful extraction of a well-positioned UAC. This case highlights that serious ischemic complications are not confined to catheter dwell time or malposition and may be precipitated by removal itself in the presence of immature collateral circulation and transient coagulopathy.

## Introduction

Umbilical artery catheters (UACs) are commonly placed in extremely preterm neonates requiring admission to the neonatal intensive care unit, with approximately half of infants born before 29 weeks' gestation receiving UAC placement [1]. UACs enable reliable arterial blood gas sampling, continuous blood pressure monitoring, and safe administration of medications in preterm neonates, and these functions are essential for the management and stabilization of the most critically ill infants, such as those admitted to our level IV neonatal intensive care unit. Although thromboembolic and ischemic complications are well-recognized risks, occurring in approximately 4% of neonates with severe manifestations, they are usually attributed to catheter malposition, vasospasm, or thrombosis during dwell time [2,3]. Because the catheter tip lies within the descending aorta, vascular complications predominantly affect distal arterial territories supplying the pelvis, lower extremities, and abdominal organs (manifesting as renal and mesenteric ischemia) and, rarely, isolated gluteal necrosis [2,4-6]. Serious ischemic injury occurring or progressing after recognition of early warning signs and uncomplicated removal of a UAC is uncommon. In addition, there is little reported in the literature on isolated perianal necrosis following UAC extraction when the tip position was radiologically confirmed throughout use, and the catheter was verified to be intact on removal [7]. This presentation indicates likely decreased perfusion of the inferior rectal branches of the internal pudendal artery, which arise from the internal iliac circulation and supply the perianal region. This case highlights that clinically significant perfusion-related events can present even after correct catheter placement and uneventful removal, emphasizing the need for continued vigilance in extremely preterm infants who may have underlying coagulopathy, heightened vascular fragility, and underdeveloped anastomoses.

## Case presentation

**Figure 1:**
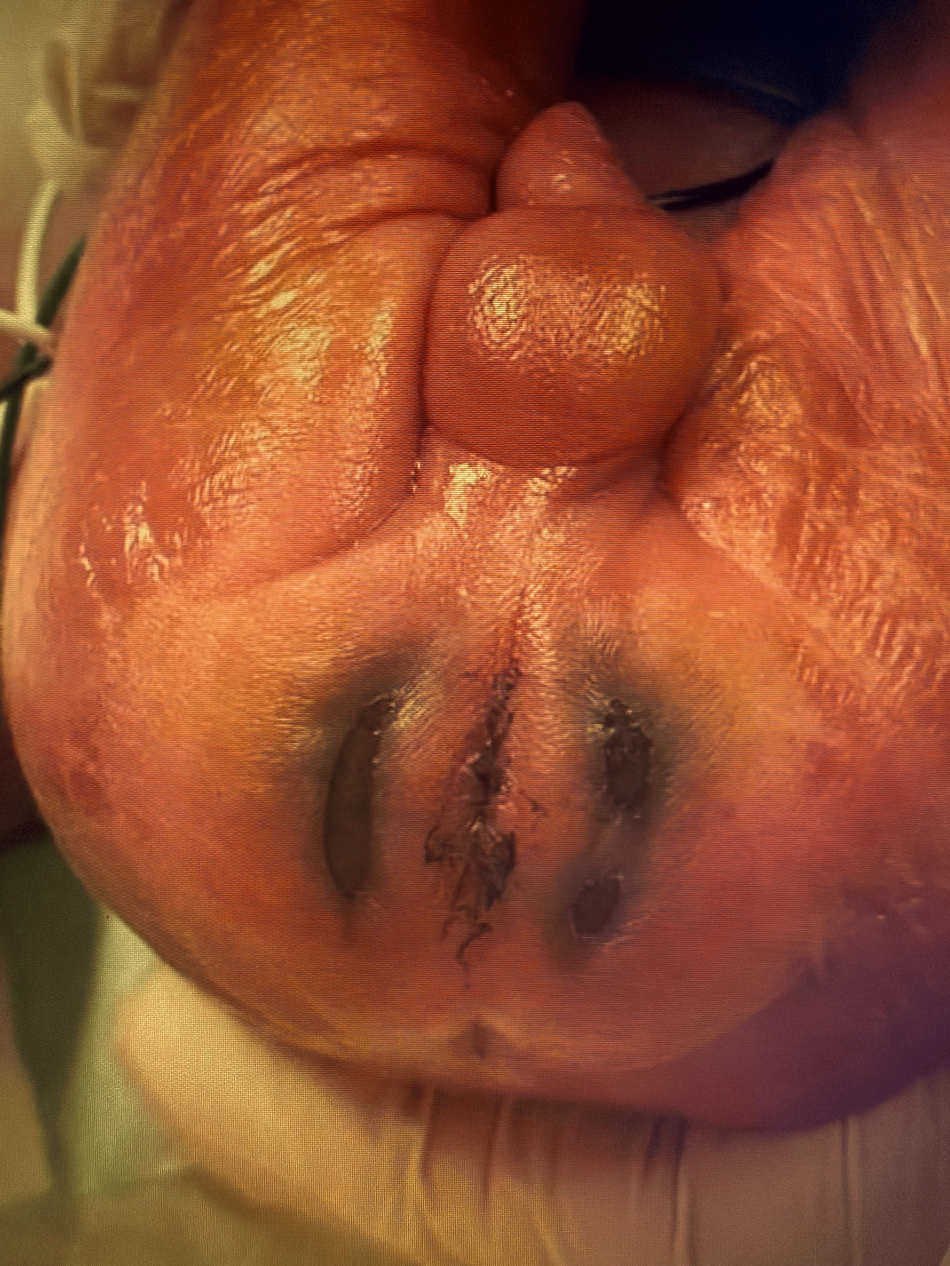
Bilateral crescent-shaped areas of necrosis about 3 x 1 cm with overlying blistering on day 13 of life.

**Figure 2:**
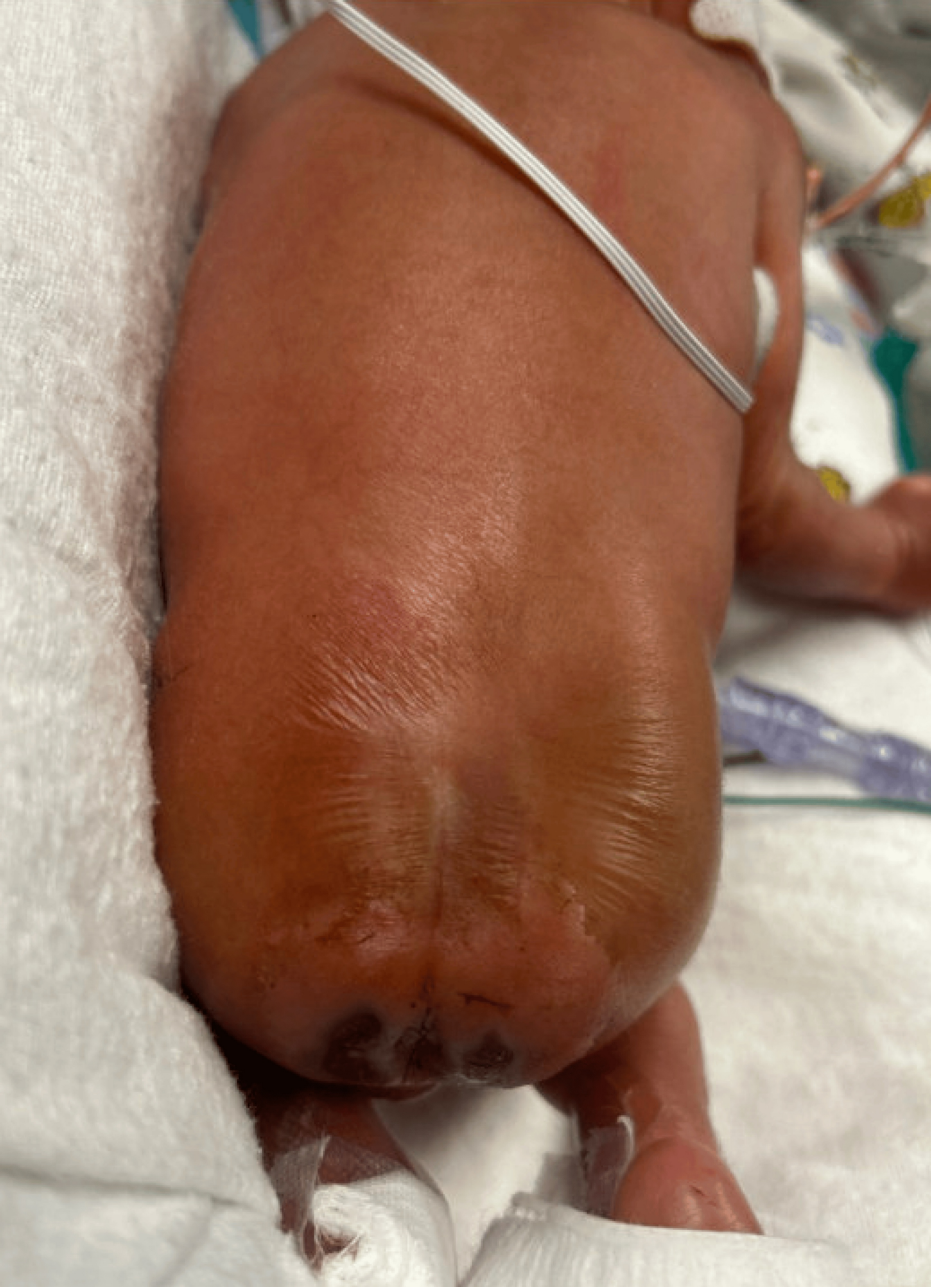
Discoloration encompassing the lower back and gluteal cleft following removal of the umbilical arterial catheter.

**Figure 3:**
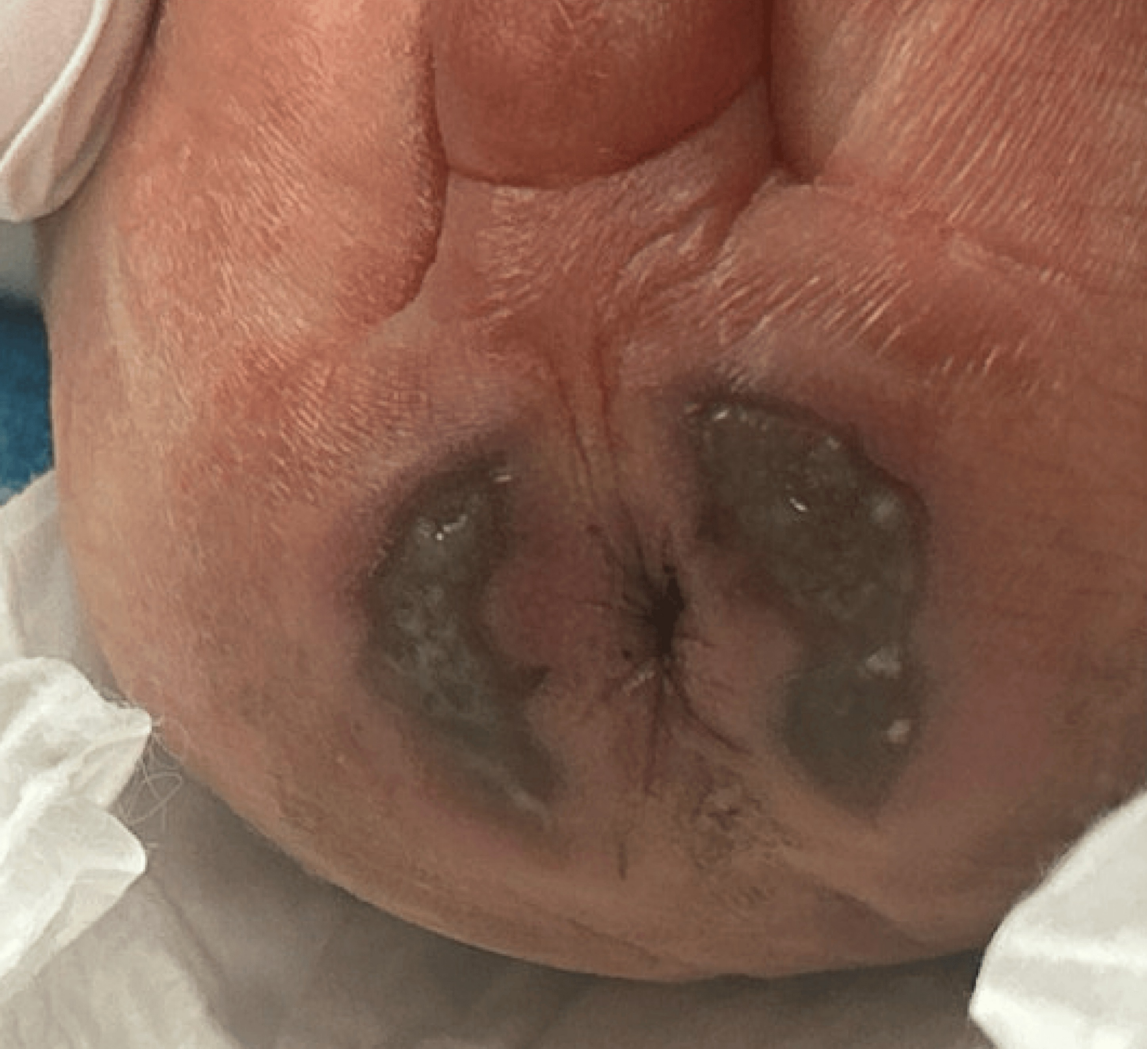
Perianal necrosis following umbilical arterial catheter removal at a maximum measured area of 3.5 × 2 cm.

A 25-week gestational age male neonate on day 0 of life was admitted to the NICU for prematurity and respiratory failure following emergency cesarean delivery. Intubation was required, and both UAC and umbilical venous catheter (UVC) were inserted with imaging confirming proper end placement. Infant health soon improved, and on day six of life, the UAC was removed intact, with the insertion depth verified at 11 cm. Upon routine examination approximately 4-6 hours after uncomplicated UAC removal, bilateral faint erythematous-to-bluish macular discoloration was noted in the perianal and gluteal cleft regions, without initial blanching on pressure or associated swelling. UVC was removed as a result. Over the next 72 hours, skin color continued to deteriorate, and the discoloration progressed to well-demarcated, non-blanching violaceous patches with early bullae formation localized to the perianal region. On day 13 of life, hematology-oncology and plastic surgery were consulted for suspected protein C deficiency and wound care. Examination disclosed bilateral crescent-shaped necrotic zones, approximately 3 x 1 cm each, with overlying bullae, surrounding the perianal region (Figure 1). Additionally, duskiness extended beyond the primary necrotic sites, encompassing the lower back and gluteal cleft, indicative of an ischemic wound in the distribution of the internal pudendal artery (Figure 2). The affected areas reached a maximum size of approximately 3.5 x 2 cm by day 16 of life (Figure 3).

**Figure 4:**
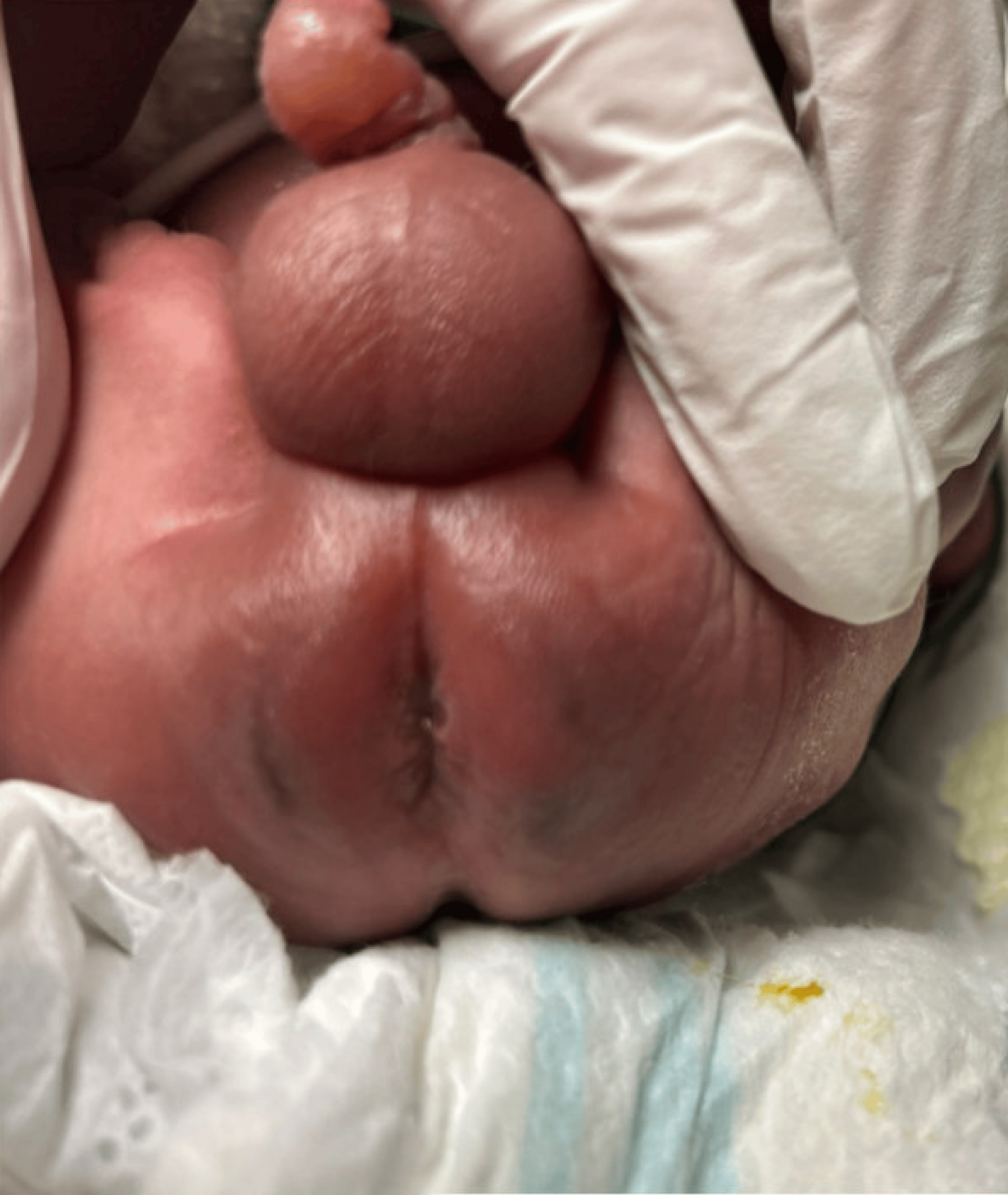
Marked interval improvement of the perianal lesion by day 28 of life, with near-complete resolution following discontinuation of Santyl.

**Figure 5:**
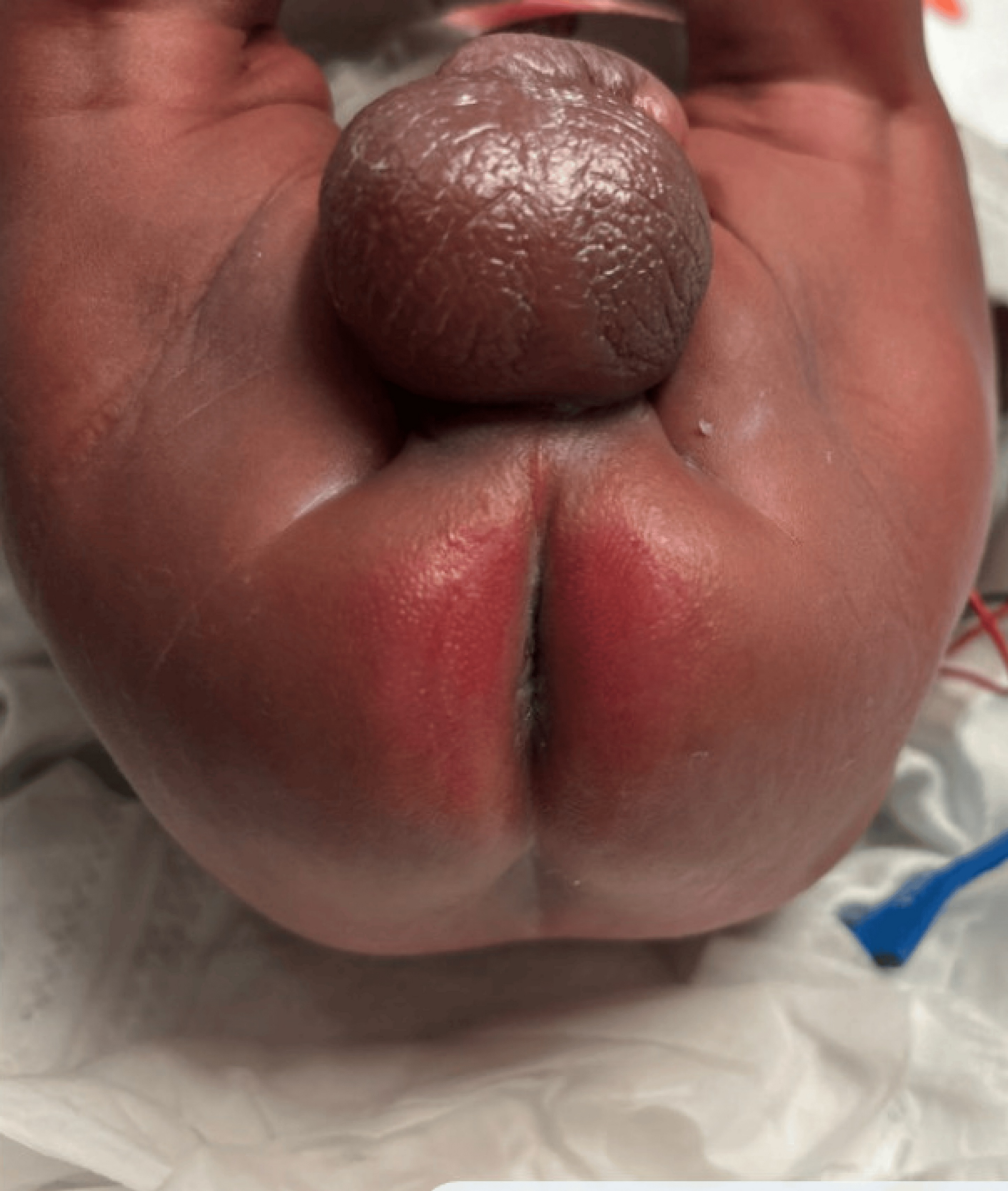
Two-month follow-up demonstrating complete resolution of the previously affected perianal area without sequelae.

Conservative wound management (Mepilex) without ointment was initiated with heparin (4 U/h). An anal speculum exam ruled out deeper tissue necrosis, and after continued improvement in wound size, daily application of Santyl (collagenase) ointment to the necrotic areas was begun on day 20 of life. By day 23 of life, perianal necrosis showed significant improvement, and central epidermolysis had resolved. Santyl therapy was discontinued thereafter, and by day 28, the wound demonstrated near-complete re-epithelialization (Figure 4). Two-month follow-up showed complete resolution of the affected area without sequelae (Figure 5). Informed parental consent was obtained for the use of clinical photographs and descriptive text for medical education purposes, with the patient’s identity protected.

## Discussion

Umbilical artery catheters (UACs) are essential in neonatal intensive care but carry a well-recognized risk of thromboembolic and ischemic complications, primarily through vasospasm, endothelial injury, or distal embolization [2,8,9]. In reported series, clinically apparent ischemia occurs in 1-5% of catheterized neonates, with gluteal or lower-limb involvement being the most common manifestation [4-6]. However, perianal ischemia following UAC use is exceptionally rare in the literature. The bilateral crescent-shaped necrotic zones with bullae in our patient mapped precisely to the angiosome of the inferior rectal branches of the internal pudendal arteries, while deeper structures, including the external anal sphincter, were spared on speculum examination. The internal pudendal artery, a terminal branch of the anterior division of the internal iliac artery, provides primary perfusion to the perianal skin and mucosa and implies partial, distal occlusion rather than complete internal iliac compromise [10].

The progression of ischemia despite prompt catheter removal after recognition of early discoloration raises several plausible mechanisms for the isolated perianal involvement observed in this case. UAC is known to cause measurable decreases in regional blood flow, with peak systolic velocities in the celiac trunk, superior mesenteric artery, renal arteries, and femoral arteries declining significantly immediately after insertion [11,12]. This may impact downstream perfusion, potentially extending to branches of the internal iliac artery, such as the internal pudendal artery. Additionally, thrombus formation along the catheter endothelium during dwell time is well documented, with associated thrombi potentially lodging in the terminal inferior rectal arteries or causing catheter-induced vasospasm in these small-caliber vessels [7,13,14]. While the primary differential diagnosis at presentation included catheter-related thromboembolic ischemia (most likely given the temporal association and vascular distribution), additional workup was begun for acquired or congenital coagulopathy, such as transient neonatal protein C/S deficiency (prompting hematology consultation), infectious causes (e.g., necrotizing fasciitis or perianal cellulitis/abscess, though absent systemic signs or fever argued against), and less likely non-accidental injury or pressure-related necrosis. Protein C and S activity was assayed and found to be within expected neonatal ranges (accounting for gestational age-adjusted norms). Fibrinogen (263 mg/dL) and platelet count (235,000/mm³) at birth were normal, while prothrombin time (16.0 s) and partial thromboplastin time (43.0 s) were only mildly prolonged, consistent with the physiological immaturity of the coagulation system in a preterm infant. Negative blood and wound cultures, together with the precise angiosomal distribution corresponding to inferior rectal artery branches, supported an ischemic rather than primarily infectious or coagulation etiology.

Adherence to established guidelines for umbilical artery catheterization is essential to minimize ischemic complications. High-position tip placement (T6-T10 in the descending thoracic aorta, above the origins of the celiac, superior mesenteric, and renal arteries) is associated with fewer vascular complications compared with low positioning and should be confirmed radiographically immediately after insertion [8,15]. Current guidelines also recommend low-dose heparin (0.25-1 U/mL in the infusate) during UAC dwell to maintain patency and reduce the risk of occlusion, though evidence to prevent clinical ischemia is limited [16]. Following removal, infants require heightened vigilance with serial examination of the gluteal, perianal, and lower-extremity skin, as ischemic changes can manifest or progress rapidly even after correct positioning and intact catheter extraction. Although current guidelines do not specify a post-removal monitoring duration, clinical practice recommends frequent skin assessments (e.g., every 2-4 hours) for at least 24 hours, as a subclinical thrombosis may be present. For UAC-associated ischemia, prompt catheter removal is essential, followed by conservative management including systemic heparin for thrombosis, topical nitroglycerin for vasospasm, supportive wound care, and escalation to thrombolysis or surgery in severe cases. Conservative management with local dry wound care (Mepilex) and low-dose systemic heparin (4 U/h) in this case achieved near-complete re-epithelialization by day 28 without surgical debridement or long-term sequelae, which is consistent with successful non-operative strategies reported in similar gluteal necrosis cases, where prompt catheter removal, anticoagulation, and supportive care salvaged tissue without amputation [2,4,6,7].

## Conclusions

In summary, this case illustrates a rare manifestation of UAC-associated ischemia, where isolated perianal necrosis progressed despite early recognition of discoloration and prompt removal of a well-positioned catheter. To our knowledge, this represents a novel instance of such severe, bilateral crescent-shaped perianal necrosis in the distribution of the internal pudendal artery's inferior rectal branches, highlighting a critical knowledge gap in the functional maturity of neonatal collateral anastomoses and underscoring the unique vulnerabilities of extremely preterm infants, such as immature vascular regulation and transient coagulopathy. Clinicians should maintain heightened vigilance post-UAC extraction, especially in high-risk populations such as premature infants, and consider urgent hematological evaluation, perfusion assessment, and low-dose heparin when new dusky skin changes appear, as timely conservative management can achieve complete resolution without surgical intervention.
